# Upregulation of Inflammatory Genes and Downregulation of Sclerostin Gene Expression Are Key Elements in the Early Phase of Fragility Fracture Healing

**DOI:** 10.1371/journal.pone.0016947

**Published:** 2011-02-11

**Authors:** Joana Caetano-Lopes, Ana Lopes, Ana Rodrigues, Diana Fernandes, Inês P. Perpétuo, Teresa Monjardino, Raquel Lucas, Jacinto Monteiro, Yrjö T. Konttinen, Helena Canhão, João E. Fonseca

**Affiliations:** 1 Rheumatology Research Unit, Faculdade de Medicina da Universidade de Lisboa, Instituto de Medicina Molecular, Lisbon, Portugal; 2 Serviço de Reumatologia e Doenças Ósseas Metabólicas, Hospital de Santa Maria, Lisbon, Portugal; 3 Department of Hygiene and Epidemiology, University of Porto Medical School, Porto, Portugal; 4 Institute of Public Health, University of Porto, Porto, Portugal; 5 Orthopaedics Department, Hospital de Santa Maria, Lisbon, Portugal; 6 Department of Medicine, University of Helsinki, Helsinki, Finland; 7 ORTON Orthopaedic Hospital of the Invalid Foundation, Helsinki, Finland; 8 COXA Hospital for Joint Replacement, Tampere, Finland; Instituto Nacional de Câncer, Brazil

## Abstract

**Background:**

Fracture healing is orchestrated by a specific set of events that culminates in the repair of bone and reachievement of its biomechanical properties. The aim of our work was to study the sequence of gene expression events involved in inflammation and bone remodeling occurring in the early phases of callus formation in osteoporotic patients.

**Methodology/Principal Findings:**

Fifty-six patients submitted to hip replacement surgery after a low-energy hip fracture were enrolled in this study. The patients were grouped according to the time interval between fracture and surgery: bone collected within 3 days after fracture (n = 13); between the 4^th^ and 7^th^ day (n = 33); and after one week from the fracture (n = 10). Inflammation- and bone metabolism-related genes were assessed at the fracture site. The expression of pro-inflammatory cytokines was increased in the first days after fracture. The genes responsible for bone formation and resorption were upregulated one week after fracture. The increase in RANKL expression occurred just before that, between the 4^th^–7^th^ days after fracture. Sclerostin expression diminished during the first days after fracture.

**Conclusions:**

The expression of inflammation-related genes, especially IL-6, is highest at the very first days after fracture but from day 4 onwards there is a shift towards bone remodeling genes, suggesting that the inflammatory phase triggers bone healing. We propose that an initial inflammatory stimulus and a decrease in sclerostin-related effects are the key components in fracture healing. In osteoporotic patients, cellular machinery seems to adequately react to the inflammatory stimulus, therefore local promotion of these events might constitute a promising medical intervention to accelerate fracture healing.

## Introduction

The management of fragility fractures associated with osteoporosis is difficult due to several factors including inadequate fixation strength of implants used to stabilize the fracture until union of bone occurs. In particular, the fragility fractures affecting the metaphyseal region of long bones are associated with an increased rate of complications. Several studies report nonunion in 2–10%, malalignment after surgery in 4–40%, metal work failure in 1–10%, and reoperation in 3–23% [1]. Experimental studies have shown that the decline in the capacity for fracture repair is age related. Disturbance of the full redevelopment of mechanical strength within fracture calluses in elderly animals has been shown in experimental rat models. In the human being it is possible that fracture healing is affected by aging [2], particularly in the elderly osteoporotic patients [3]. In another study it was described that in 6, 26 and 52 weeks old rats, there is upon aging a delay in radiographic progression of fracture healing but the expression of the key genes involved in this process is not age-dependent [1], [4], [5]. The fracture healing response and its temporal gene expression in elderly patients with osteoporosis has not been adequately investigated at the cellular and molecular level. Identification of the mechanisms that lead to fracture healing disturbances in patients with osteoporosis is of outstanding importance because they could allow prevention and better management of these healing complications. In addition, the biological processes behind fracture healing in osteoporosis might hold the key for future medical interventions.

Fracture healing recapitulates certain aspects of skeletal development and growth, involving interplay of cells, growth factors and extracellular matrix. Following injury, a blood clot is formed in the fracture site [6], [7]. This hematoma is the source of several signaling molecules that induce an inflammatory cascade of events that initiate healing [8], [9]. Based on histological observations of healing fractures, bone repair was defined in animal models by an initial inflammatory phase (lasting for about three days), a catabolic phase where damaged tissues are removed, and an anabolic phase where new bone is rebuilt. Within several days of the initial inflammatory response there is a sequence of events that results in the formation of new bone through the development of a structure named callus. Experimental studies have related temporal gene expression with bone healing. In a study with Sprague-Dawley rats, gene expression was evaluated on days 3 and 11 post-fracture. The authors showed that different molecular pathways of gene expression regulate different phases of bone healing [10].

This work aims to study the profile of genes involved in inflammation and bone remodeling during the 3 major steps of the early phase of callus formation in human bone after a hip fragility fracture.

## Results

### Study population

Fifty-six patients 80±7 years of age, 75% of female gender, which suffered a hip fragility fracture, were enrolled in this study. There were no statistical significant differences in age and gender between the 3 study groups (Table 1): those who had surgery less than three days after fracture (group 1), between four and seven days (group 2) and eight or more days post-fracture (group 3).

**Table 1:** Description of the study population divided between the event of fracture and surgery.

	Group 1	Group 2	Group 3	p-value
	Until 3 days post-fracture	4 to 7 days post-fracture	8 or more days post-fracture	
Number of patients	13	33	10	
Age (years)	80±5	79±7	83±7	0.326
Female gender (%)	85	79	50	0.121

Values represent mean±standard deviation.

Comparisons between groups performed with ANOVA or chi-square tests.

### Inflammation and growth factors genes

**Figure 1:**
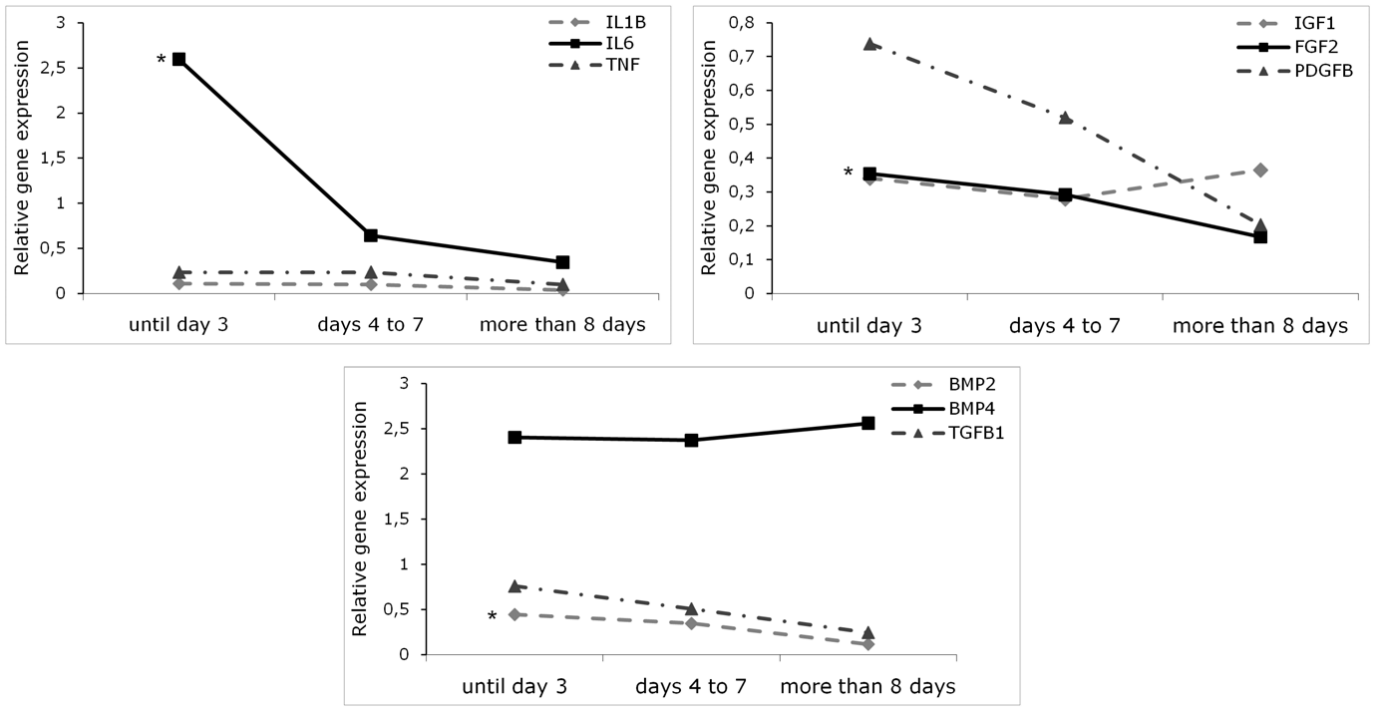
Relative expression of inflammation and growth factors genes grouped according to the time between the event of fracture and the surgery. Each gene was normalized to the expression of the housekeeping genes *B2M* and *PMM1*. *p-value<0.05 for comparisons between the three groups. (Points represent median values). IL1B – interleukin-1β; IL6 – interleukin-6; TNF – tumor necrosis factor; IGF1 – insulin growth factor-1 ; FGF2 – fibroblast growth factor 2 ; PDGFB – platelet derived growth factor β; BMP – Bone morphogenetic protein; TGFB1 – transforming growth factor β1.

The local expression of nine genes related to the inflammatory phase of bone healing (IL-1β, IL-6, TNF, BMP2, BMP4, TGF-β1, IGF-I, FGF-2 and PDGF-β) was analyzed (Figure 1, Table 2). IL-1β, IL-6 and TNF are cytokines that have an important role in potentiating the inflammatory cascade. Concordantly, the expression of these genes was highest during the first 3 post-fracture days and decreases thereafter. Specifically, *IL6* had a higher expression in group 1 than in group 2 (p-value = 0.021) and *IL1B*, although expressed at low levels, remained stable, decreasing slightly after the 4^th^–7^th^ day post-fracture (p-value = 0.087). On the contrary, *TNF* expression was stable, showing only a slight tendency to decrease over time (p-value = 0.208).

**Table 2:** Comparison between the relative gene expression levels of patients submitted to hip replacement surgery due to low-energy fracture in relation to the days between fracture and surgery.

	Fragility fracture patients			p-value*
	Until 3 days post-fracture	4–7 days post-fracture	8 or more days post-fracture	
***IL1B***	0.111(0.07–0.30)	0.101(0.03–0.16)	0.039(0.02–0.11)	0.087
***IL6***	2.596(0.94–5.23)	0.643(0.24–2.46)	0.348(0.08–0.90)	0.021
***TNF***	0.236(0.20–0.63)	0.238(0.09–0.43)	0.102(0.08–0.56)	0.208
***TGFB1***	0.759(0.28–0.94)	0.507(0.25–0.83)	0.244(0.17–0.49)	0.051
***BMP2***	0.443(0.20–1.32)	0.344(0.09–0.55)	0.114(0.03–0.41)	0.023
***BMP4***	2.406(1.16–6.66)	2.372(0.72–5.54)	2.516(0.86–3.26)	0.852
***IGFI***	0.341(0.13–2.24)	0.280(0.16–0.80)	0.365(0.27–0.66)	0.817
***FGF2***	0.354(0.25–1.08)	0.292(0.12–0.85)	0.168(0.10–0.36)	0.091
***PDGFB***	0.738(0.30–1.71)	0.520(0.19–0.82)	0.203(0.08–0.58)	0.043
***OPG***	3.340(1.63–4.56)	1.938(0.99–4.18)	0.979(0.85–2.09)	0.168
***RANKL***	13.803(5.46–22.36)	25.838(8.11–39.20)	15.305(3.86–32.70)	0.267
***RANK***	0.095(0.06–0.37)	0.290(0.14–0.52)	0.438(0.24–0.86)	0.072
***RANKL/OPG***	4.887(2.71–7.78)	11.362(5.32–22.28)	5.581(3.08–23.08)	0.078
***CBFA1/RUNX2***	1.954(1.08–2.63)	2.574(1.31–4.02)	2.178(1.12–2.58)	0.521
***OSX***	0.629(0.28–1.86)	0.655(0.24–0.87)	0.888(0.68–2.71)	0.149
***ALP***	5.393(2.53–7.86)	4.407(2.47–5.95)	4.054(2.64–10.05)	0.726
***SOST***	1.402(1.07–3.66)	0.668(0.39–1.18)	0.274(0.19–0.77)	0.001
***TRAP***	4.204(1.31–11.46)	9.660(3.78–24.41)	22.758(9.18–83.48)	0.009
***CTSK***	30.276(8.72–132.81)	63.593(15.67–250.62)	151.91(87.71–558.88)	0.027
***ITGB3***	2.045(1.43–2.97)	1.619(1.16–2.79)	1.292(0.82–2.48)	0.658
***ATP6V0D2***	4.898(2.78–10.71)	9.839(4.51–25.20)	32.378(7.65–68.59)	0.076

Values represent median (Q1–Q3).

Comparisons between the 3 groups performed with Kruskall-Wallis H test.

*p-value for comparison between the 3 groups.

IL1B – interleukin-1β; IL6 – interleukin-6; TNF – tumor necrosis factor; TGFB1 – transforming growth factor β1; BMP – Bone morphogenetic protein; IGF1 – insulin growth factor-1; FGF2 – fibroblast growth factor 2; PDGFB – platelet derived growth factor β; OPG – osteoprotegerin; RANK – receptor activator of nuclear factor κB; RANKL – RANK ligand; CBFA1/RUNX2 – core binding factor α1/runt-related transcription factor 2; OSX – osterix; ALP – alkaline phosphatase; SOST – sclerostin; TRAP – tartrate-resistant acid phosphatase; CTSK – cathepsin K; ITGB3 – subunit β3 of the integrin αvβ3; ATP - ATPase H^+^ transporter.

BMPs are a set of growth factors and cytokines belonging to the TGF-β superfamily and are involved in the creation of bone tissue architecture. In fracture healing, BMP-2 and BMP-4 play important roles in osteoblast differentiation. Accordingly, it was observed that the expression levels of *BMP2* were highest until 3 days post-fracture and decreased thereafter (p-value = 0.023), while *BMP4* expression remained fairly constant in all groups (p-value = 0.852). *TGFB* exhibited a constant negative slope between the three groups (p-value = 0.051).

IGF-I is a hormone involved in bone matrix synthesis and there were no differences in its expression levels in the three groups analyzed (p-value = 0.817). The growth factors FGF-2 and PDGF-β are involved in the formation of new blood vessels. Their expression tended to decrease slightly from group 1 to group 2 and was clearly decreased after 8 days post-fracture (*FGF2*: p-value = 0.091 and *PDGFβ*: p-value = 0.043).

Overall, these findings suggest that the expression levels of inflammatory genes and growth factors are particularly high during the three first days post-fracture and decrease from the day 4 onwards.

### Osteoprotegerin, RANK and RANKL

**Figure 2:**
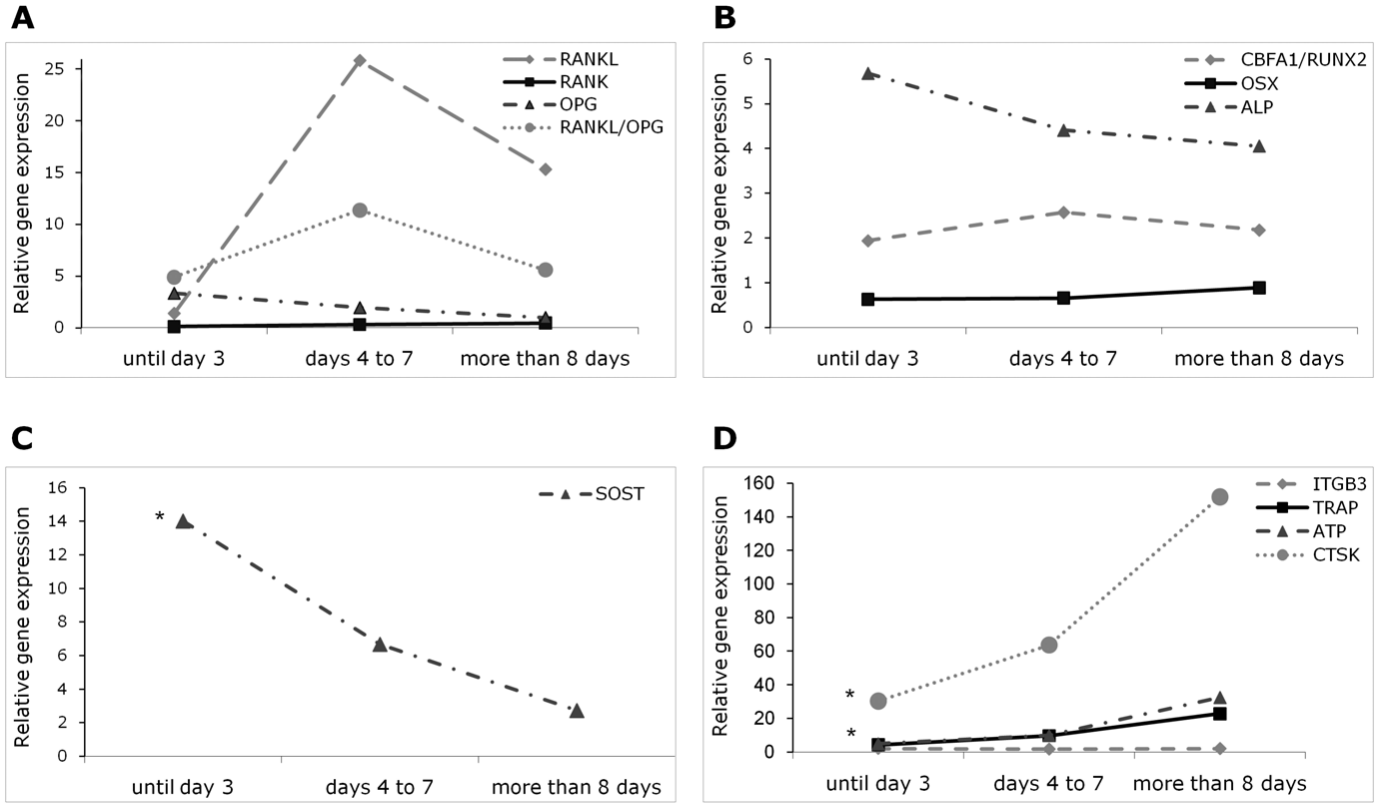
Relative expression of bone metabolism-related genes divided according to the time between the event of fracture and the surgery. *RANK*, *RANKL* and *OPG* (**A**), osteoblast (**B**), osteocyte (**C**) and osteoclast-specific genes (**D**) were studied in the three study groups. Each gene was normalized to the expression of the housekeeping genes *B2M* and *PMM1*. *p-value<0.05 for comparisons between the three groups. (Points represent median values). RANK – receptor activator of nuclear factor κB; RANKL – RANK ligand; OPG – osteoprotegerin; CBFA1/RUNX2 – core binding factor α1/runt-related transcription factor 2; OSX – osterix; ALP – alkaline phosphatase; SOST – sclerostin; ITGB3 – subunit β3 of the integrin αvβ3; TRAP – tartrate-resistant acid phosphatase; ATP - ATPase H^+^ transporter; CTSK – cathepsin K.

OPG is a negative regulator of bone resorption and, as expected, its expression was slightly lower in group 3 than in group 1 (p-value = 0.168) (Figure 2A, Table 2). On the other hand, *RANK* produced by osteoclast precursors showed a tendency to increase over time (p-value = 0.072). Finally, *RANKL* expressed by osteoblasts, stromal cells and immune system cells had its highest level at days 4 to 7 post-fracture (group 2) and decreased thereafter (p-value = 0.267).

The ratio RANKL/OPG regulates the balance between remodeling and formation. In this study, the ratio *RANKL*/*OPG* mRNA peaked in group 2 and tended to decrease later on (p-value = 0.078).

### Steoblast-related genes

The expression of three genes that play important functions in the osteoblast and its activity was studied (Figure 2B, Table 2). Core-binding factor, alfa subunit 1/runt-related transcription factor 2 (CBFA1/RUNX2) and osterix (OSX) are transcription factors that play a crucial role in osteoblast differentiation, and ALP is an enzyme expressed at a later stage being involved in bone matrix maintenance. In our results, *CBFA1/RUNX2* expression was slightly higher in group 2 than in group 1 and then remained constant and was similar in groups 2 to 3 (p-value = 0.521). On the other hand, *OSX* expression levels were similar in groups 1 and 2 and marginally higher in group 3 (p-value = 0.149). Regarding *ALP*, levels were slightly lower in group 2 as compared to group 1 but were similar between groups 2 and 3 (p-value = 0.726).

### Osteocyte-related genes

Sclerostin (SOST) is produced by the osteocyte and regulates negatively osteoblast differentiation by inhibiting Wnt/β-catenin signaling. In this work we found a highly significant reduction in *SOST* levels overtime (p-value = 0.001) (Figure 2C, Table 2).

### Osteoclast-related genes

Four genes that regulate osteoclast differentiation and function were studied (Figure 2D, Table 2). Tartrate-resistant acid phosphatase (TRAP) is an enzyme expressed by pre-osteoclasts and active osteoclasts; on the other hand, cathepsin K (CTSK), β3 subunit of the αvβ3 integrin (ITGB3) and ATPase H^+^ transporter (ATP6V0D2) are proteins involved in the process of bone resorption.

We observed a marked increase in *TRAP* and a more modest change in *CTSK* and *ATP6V0D2* expression over the post fracture period (*TRAP*: p-value = 0.009; *CTSK*: p-value = 0.027 and *ATP6V0D2*: p-value = 0.076). On the other hand, the expression of *ITGB3* remained constant (p-value = 0.658).

## Discussion

Although some studies have addressed the sequence of events in fracture healing, the research was mainly based on histological examination of healthy individual's tissue and on molecular studies in animal models. Based on these previous histological and molecular studies of healing fractures the initial stages of these process have been proposed to include an early inflammatory and unspecific anabolic phase (first 24 hours up to third day), immediately followed by a non specific catabolic phase (up to the end of the first week) that sets the conditions for a more bone specific anabolic phase (first week and thereafter) [10], [11], [12], [13], [14]. Thus, in our study, patients were grouped into these three phases according to the time between fracture and surgery and the main objective was to address the gene expression variations during early callus formation in patients that have suffered a hip fragility fracture. We showed that many inflammation-related genes have higher levels of expression until 3 days post-trauma while genes related to osteoblast and osteoclast activity increase at day 4 and thereafter.

Cytokine gene expression (*IL1B*, *IL6* and *TNF*) was more pronounced during the first days after fracture, as described in younger individuals. Specifically, the decrease in *IL6* expression levels was far more pronounced than what was observed with the other pro-inflammatory cytokines evaluated. Of interest, reports state that *IL1B* is upregulated in response to the fracture event but in *IL1B* knockout mice there was no change in callus formation and bone and cartilage matrix production [15]. On the other hand, in the absence of TNF signaling there was a 2–4 days delay in chondrogenic differentiation and a 2–3 weeks delay in endochondral tissue resorption [16]. Regarding IL-6, studies in knockout mice have shown that there was a delay in callus formation and lower osteoclast density [17]. Therefore, in accordance with our results, IL-6 appears to have a pivotal role in the early phase of fracture healing, probably through the increase in the pro-osteoclastogenic stimuli. Moreover, expression levels of *TGFB*, *BMP2*, *BMP4*, *PDGFB* and *FGF2* was highest during the first 3 days post-fracture. The variation encountered in these elderly fragility fracture patients is similar to the findings obtained in animal models studies [8] and in healthy younger subjects [18] where the inflammatory phase occurs before day 3 post-fracture, being IL-6 a crucial player in this early phase of fracture healing.

Regarding the RANK-RANKL-OPG system, *OPG* expression diminished gradually after fracture, releasing the inhibitory signal for osteoclast differentiation. Concordantly, *RANKL* peaks at 4^th^–7^th^ day after trauma, creating a stimulus for osteoclast differentiation from its precursors. Therefore, the ratio *RANKL*/*OPG* was high during days 4–7 post-fracture, not only due to an increase in *RANKL* but also to a decrease in *OPG* expression switching the balance to a pro-resorptive status, as described in a young mouse model [9].

Concerning osteoblast differentiation and activity, it was observed that *CBFA1/RUNX2* and *OSX*, two regulatory factors essential for its differentiation [19], had a weak increase indicating the beginning of an initial osteogenic phase. *CBFA1/RUNX2* increases from the initial phase of bone healing whereas *OSX* increases after 4 days post-fracture sustaining the evidence that *OSX* acts later than *CBFA1/RUNX2* in osteoblast lineage commitment [20]. On the other hand, *ALP*, a marker for osteoblast activity, decreases from early stages of bone healing. This gene expression profile had been already observed in other studies [18], [21], [22] and it is not entirely surprising, since this enzyme is involved in bone matrix production and our study is focused on the early changes related to fracture, in a stage where the formation of new bone matrix is still not occurring.

Sclerostin is a protein produced by the osteocyte that inhibits canonical Wnt/β-catenin signaling, thus blocking osteoblast proliferation and differentiation. The contribution of this pathway to fracture healing depends on the function of β-catenin in different stages of fracture repair, namely in the commitment and regulation of osteoblasts [23]. Only one study in young mice has addressed the levels of expression of sclerostin during fracture repair and they found that this protein was downregulated during the process [24]. In fact, our results, the first obtained in fragility fracture patients, showed that *SOST* expression decreases significantly from the beginning of the healing cascade, suggesting that there is an initial blockage of osteoblast proliferation and differentiation that is subsequently released over the period of fracture healing.

The role of the osteoclast in bone healing is somewhat controversial. Bone formation overcomes the loss of continuity and osteoclasts seem to play a role at a later phase, in the remodeling stage. Moreover, in a longitudinal study where the serum levels of biochemical markers associated with bone metabolism were assessed, the authors showed that the markers for bone resorption remained elevated up to four months after fracture [21]. At gene expression level, we found that the osteoclast-specific genes *TRAP*, *CTSK* and *ATP6V0D2* were significantly increased from day 8 onward after fracture, pointing to an activation of osteoclast function. In fact, the RANKL/OPG ratio is highest in group 2, whereas the CTSK values are increased in group 3, indicating that during 4–7 days after fracture, osteoclastogenesis stimulus was ongoing intensively whereas at day 8 and later osteoclasts containing cathepsin K had already been formed in relatively high numbers. The active role of osteoclast during the early phase of fracture healing was already described in sheep where it was proposed that these cells not only resorb bone but adjust the system, together with osteoblasts, in order to improve bone strength [25].

Due to the fact that we are dealing with human subjects, the study had to have a cross-sectional design. Thus, it is not possible to rule out the intrinsic variability of different individuals. However, the statistical significance for many of the changes described seems to refute this. Besides, the RNA used was extracted from the site of fracture (trabecular bone) that not only has the bone cells that we are interested in, but also other cell types, such as adipocytes, bone marrow cells and cells infiltrating the tissues during the initial healing phase. However, the bone remodeling genes studied are relatively specific for bone cells and it is unlikely that this technical aspect represent a relevant confounding factor in our study.

Taken together, our results indicate that in patients with hip fragility fractures, the expression of inflammation-related genes is highest during the first days after fracture but from day 4 onwards there is a shift towards bone cell remodeling genes, suggesting that the machinery of bone healing is conserved in osteoporotic bone.

In addition, the changes observed in IL-6 expression profile suggest that this pro-inflammatory cytokine plays a pivotal role in triggering the healing cascade. Moreover, sclerostin expression is quickly reduced after fracture and we hypothesize that this allows osteoblasts to escape from its inhibitory effect, thus promoting the expression of bone formation genes. Interestingly, RANKL expression is subsequently increased, generating the stimulus for osteoclast activity, as confirmed also by the later rise in the expression of the bone resorption-related genes. Our findings bring new insights for clarifying bone fracture healing process in osteoporotic patients. We propose that an initial inflammatory stimulus and a decrease in sclerostin-related effects are key events for an adequate fracture healing. Thus, in osteoporotic patients, locally promoting these events might provide promising medical interventions for accelerating fracture healing and reduce the rate of complications.

## Methods

### Sample collection

Patients that suffered a low-energy hip fracture and underwent total hip replacement surgery at the Orthopedic Department of Hospital de Santa Maria were consecutively recruited for this study from 2007 until 2009. Epidemiological and clinical data such as age, gender and days between the fracture and surgery were collected. Patients with other metabolic bone diseases and with bone metastases were excluded.

Written informed consent was obtained from all patients and the study was conducted in accordance with the ethical principles for medical research involving human subjects expressed in the Declaration of Helsinki, as amended in Edinburgh (2000), and was approved by Santa Maia Hospital Ethics Committee.

According to the time between fracture and surgery, patients were divided in three groups: those who had hip replacement surgery between zero and three days after fracture (group 1), four and seven days after fracture (group 2) or eight or more days after fracture (group 3).

After the medical procedure, the femoral epiphyses were snap-frozen at −80°C.

### RNA extraction

Without defrosting the bone specimen, small trabecular bone pieces were collected from the site of fracture and pulverized using a mortar and pestle. RNA was then extracted using TRIzol reagent (Invitrogen, UK) with proteinase K (Bioline, UK) digestion [26] to better dissolve the dense extracellular matrix.

The procedure used was a modified version of the protocol described by Ireland [27]. Briefly, 80mg of bone powder was placed in TRIzol reagent and homogenized. Lipids were solubilized with 0.2 volumes of chloroform and the fraction containing RNA was preserved. Proteinase K digestion (3.3µg proteinase K/mg bone) was performed at 55°C. RNA was precipitated with 1 volume of ice-cold isopropyl alcohol. RNA pellet was dissolved in RNase/DNase-free water. As this method leaves residual chemical contaminants, RNA was cleaned using a commercial kit (RNeasy mini kit, Qiagen, Germany) and genomic DNA contaminants were removed by DNaseI treatment (Qiagen, Germany). RNA concentration was determined spectrophotometrically (Nanodrop ND-1000 Spectrophotometer, Thermo Fisher Scientific, USA) and its integrity was assessed by lab-on-a-chip technology (Agilent RNA 6000 Nano Kit, Agilent technologies, USA) according to the manufacturer's instructions. RNA was stored at −80°C until further use.

### Quantitative reverse transcription-polymerase chain reaction (PCR)

Reverse transcription cDNA synthesis was performed on 60ng of RNA from each sample using the DyNAmo cDNA synthesis kit (Finnzymes, Finland) and 300ng of random hexamer primers according to the manufacturer's instructions.

Each cDNA template (3ng/µl) was amplified in duplicate with DyNAmo Flash SYBR green qPCR kit (Finnzymes, Finland) on a Rotor-Gene thermocycler (Qiagen, Germany) according to the manufacturer's instructions. Reactions were incubated at 50°C for 2 minutes and at 95°C for 7 minutes, followed by denaturation at 95°C for 10 seconds and annealing/extension at 60°C for 10 seconds. The reactions were validated by the presence of a single peak in the melt curve analysis.

Primers for the housekeeping and target genes (Table 3) were designed using the software *Probefinder* (http://qpcr.probefinder.com, Roche, Switzerland) in order to anneal in separate exons preventing amplification of contaminating genomic DNA.

**Table 3:** Real time PCR primer sequences of the genes studied.

Gene	GenBank number	Primer sequences
*B2M*	NM_004048	F: CTATCCAGCGTACGCCAAAGATTC
		R: CTTGCTGAAAGACAAGTCTGAATG
*PMM1*	NM_002676	F: GAATGGCATGCTGAACATCT
		R: TCCCGGATCTTCTCTTTCTTG
*IL1B*	NM_000576	F: TACCTGTCCTGCGTGTTGAA
		R: TCTTTGGGTAATTTTTGGGATCT
*IL6*	NM_000600	F: GATGAGTACAAAAGTCCTGATCCA
		R: GATGAGTACAAAAGTCCTGATCCA
*TNF*	NM_000594	F: CAGCCTCTTCTCCTTCCTGAT
		R: GCCAGAGGGCTGATTAGAGA
*TGFB1*	NM_000660	F: GCAGCACGTGGAGCTGTA
		R: CAGCCGGTTGCTGAGGTA
*BMP2*	NM_001200	F: CGGACTGCGGTCTCCTAA
		R: GGAAGCAGCAACGCTAGAAG
*BMP4*	NM_001202	F: CTGCAACCGTTCAGAGGTC
		R: TGCTCGGGATGGCACTAC
*FGF2*	NM_002006	F: TTCTTCCTGCGCATCCAC
		R: TTCTGCTTGAAGTTGTAGCTTGAT
*PDGFB*	NM_002608	F: CTGGCATGCAAGTGTGAGAC
		R: CGAATGGTCACCCGAGTTT
*IGFI*	NM_001111283	F: TGTGGAGACAGGGGCTTTTA
		R: ATCCACGATGCCTGTCTGA
*CBFA1/RUNX2*	NM_004348	F: CGGAATGCCTCTGCTGTTA
		R: TCTGTCTGTGCCTTCTGGGT
*OSX*	NM_152860	F: CATCTGCCTGGCTCCTTG
		R: CAGGGGACTGGAGCCATA
*ALP*	NM_000478	F: AGAACCCCAAAGGCTTCTTC
		R: CTTGGCTTTTCCTTCATGGT
*TRAP*	NM_001111034	F: CGGCCACGATCACAATCT
		R: GCTTTGAGGGGTCCATGA
*ITGB3*	NM_000212	F: GGGCAGTGTCATGTTGGTAG
		R: CAGCCCCAAAGAGGGATAAT
*CTSK*	NM_000396	F: GCCAGACAACAGATTTCCATC
		R: CAGAGCAAAGCTCACCACAG
*ATP6V0D2*	NM_152565	F: CATTCTTGAGTTTGAGGCCG
		R: CCGTAATGATCCGCTACGTT
*SOST*	NM_025237	F: AGACCAAAGACGTGTCCGAG
		R: GGGATGCAGCGGAAGTC
*RANK*	NM_003839	F: GAACATCATGGGACAGAGAAATC
		R: GGCAAGTAAACATGGGGTTC
*RANKL*	NM_003701	F: AGAGAAAGCGATGGTGGATG
		R: TATGGGAACCAGATGGGATG
*OPG*	NM_002546	F: CGCTCGTGTTTCTGGACAT
		R: GTAGTGGTCAGGGCAAGGG

B2M – β2-microglobulin; PMM1 - phosphomannomutase 1; IL1B – interleukin-1β; IL6 – interleukin-6; TNF – tumor necrosis factor; TGFB1 – transforming growth factor β1; BMP – Bone morphogenetic protein; IGF1 – insulin growth factor-1; FGF2 – fibroblast growth factor 2; PDGFB – platelet derived growth factor β; OPG – osteoprotegerin; RANK – receptor activator of nuclear factor κB; RANKL – RANK ligand; CBFA1/RUNX2 – core binding factor α1/runt-related transcription factor 2; OSX – osterix; ALP – alkaline phosphatase; SOST – sclerostin; TRAP – tartrate-resistant acid phosphatase; CTSK – cathepsin K; ITGB3 – subunit β3 of the integrin αvβ3; ATP - ATPase H^+^ transporter.

Real time PCR results were analyzed using the standard curve analysis. The cycle threshold (C_T_) is defined as the number of cycles required for the fluorescent signal to cross the threshold and exceed the background level. The efficiency of the PCR should be 100%, meaning that for each cycle the amount of product doubles. A good reaction should have an efficiency of 90–100%, which corresponds to a slope between −3.58 and −3.10. The conversion of the C_T_ value in relative expression levels was performed with the slope and the Y intersect extracted from the standard curve and applying the equation 10^(Y intersect-CT/slope)^ [28], [29]. The values obtained were normalized with the housekeeping genes β-2-microglobulin (*B2M*) and phosphomannomutase 1 (*PMM1*).

### Statistical analysis

To define the exposure variable, patients were divided, according to the number of days between the event of fracture and the surgery, in three groups. The distributions of continuous variables were compared between groups using either analysis of variance (ANOVA), for normally-distributed characteristics, or Kruskal-Wallis H test, for distributions with significant deviation from normality (according to Shapiro-Wilk test). For nominal variables, chi-squared test was used. Significance level was set at 0.05.

Statistical analysis was performed using the Statistical Package for Social Sciences manager software, version 17.0 (SPSS, Inc, Chicago, IL, USA).
